# External Validation of Equations to Estimate Resting Energy Expenditure in Critically Ill Children and Adolescents with and without Malnutrition: A Cross-Sectional Study

**DOI:** 10.3390/nu14194149

**Published:** 2022-10-06

**Authors:** George Briassoulis, Efrossini Briassouli, Stavroula Ilia, Panagiotis Briassoulis

**Affiliations:** 1 Pediatric Intensive Care Unit, University Hospital, School of Medicine, University of Crete, 71110 Heraklion, Greece; 2 Postgraduate Program “Emergency and Intensive Care in Children Adolescents and Young Adults”, School of Medicine, University of Crete, 71003 Heraklion, Greece; 3 Infectious Diseases Department “MAKKA”, First Department of Paediatrics, “Aghia Sophia” Children’s Hospital, National and Kapodistrian University of Athens, 11527 Athens, Greece; 4 Attikon University Hospital, School of Medicine, National and Kapodistrian University of Athens, 12462 Athens, Greece

**Keywords:** resting energy expenditure, indirect calorimetry, prediction equations, critically ill, intensive care, children, validation, accuracy, nutrition

## Abstract

We evaluated the validity of sixteen predictive energy expenditure equations for resting energy expenditure estimation (eREE) against measured resting energy expenditure using indirect calorimetry (REE_IC_) in 153 critically ill children. Predictive equations were included based on weight, height, sex, and age. The agreement between eREE and REE_IC_ was analyzed using the Bland–Altman method. Precision was defined by the 95% limits of the agreement; differences > ±10% from REE_IC_ were considered clinically unacceptable. The reliability was assessed by the intraclass correlation coefficient (Cronbach’s alpha). The influence of anthropometric, nutritional, and clinical variables on REE_IC_ was also assessed. Thirty (19.6%) of the 153 enrolled patients were malnourished (19.6%), and fifty-four were overweight (10.5%) or obese (24.8%). All patients received sedation and analgesia. Mortality was 3.9%. The calculated eREE either underestimated (median 606, IQR 512; 784 kcal/day) or overestimated (1126.6, 929; 1340 kcal/day) REE_IC_ compared with indirect calorimetry (928.3, 651; 1239 kcal/day). These differences resulted in significant biases of −342 to 592 kcal (95% limits of agreement (precision)−1107 to 1380 kcal/day) and high coefficients of variation (up to 1242%). Although predicted equations exhibited moderate reliability, the clinically acceptable ±10% accuracy rate ranged from only 6.5% to a maximum of 24.2%, with the inaccuracy varying from −31% to +71.5% of the measured patient’s energy needs. REE_IC_ (*p* = 0.017) and eREE (*p* < 0.001) were higher in the underweight compared to overweight and obese patients. Apart from a younger age, malnutrition, clinical characteristics, temperature, vasoactive drugs, neuromuscular blockade, and energy intake did not affect REE_IC_ and thereby predictive equations’ accuracy. Commonly used predictive equations for calculating energy needs are inaccurate for individual patients, either underestimating or overestimating REE_IC_ compared with indirect calorimetry. Altogether these findings underscore the urgency for measuring REE_IC_ in clinical situations where accurate knowledge of energy needs is vital.

## 1. Introduction

Accurate determination of resting energy expenditure (REE) in critically ill patients is vital because underfeeding and overfeeding are both associated with undesirable consequences. Cross-sectional and longitudinal studies have shown that mechanically ventilated children do not increase their metabolic rate during the acute phase of critical illness [1]. This finding supports the hypothesis that growth ceases during the metabolic response to critical illness or injury in children while little or no spontaneous respiratory effort or physical activity has an additional negative effect.

Predictive methods commonly used to estimate resting energy expenditure (eREE) in critically ill children are very imprecise and may lead to over- or underfeeding. Suggested equations for use in healthy children and adolescents are the Harris and Benedict [2], FAO/WHO/UNU [3], Institute for Medicine of the National Academies and Food and Nutrition Board (IOM) [4], and Schofield (height and weight, WHO) [5] equations, and the Henry (Oxford with weight and height) [6], Lawrence (Equation (3)) [7], and Kaneko [8] equations. Among the predictive equations based on age, weight, height, and sex in the pediatric population with overweight or obesity, the Dietz [9], Maffeis [10], Molnár [11], Muller [12], and Lazzer (Equation (1)) [13] equations have been reported [14]. In addition, the Mifflin equation [15] has been recently shown to be an accurate eREE equation in girls and boys without or with obesity [16]. Simplified equations reported for use in mechanically ventilated children [14,17] were the Caldwell–Kennedy equation [18], the White (Equation (2)) [19], and the Meyer (equation-C) [20]. Other equations have been either established for use in specific situations, such as anorexia nervosa or burn injuries, or were using changing indices, such as organ failures or non-standard anthropometric measurements [14]. Still, there are units using the Recommended Dietary Allowances (RDA) [21] for estimating REE in pediatric patients.

Recent nutritional guidelines recommend cautious use of estimating equations and increased surveillance for unintended caloric underfeeding and overfeeding [22]. Instead, REE should be measured by indirect calorimetry whenever possible [23,24]. Indirect calorimetry is a personalized noninvasive method that circumvents many of the problems associated with other modes of REE assessment. Since this method directly measures the conversion of energy to heat, there is no need to apply age-related, population-based data to individual critically ill children. Breath-by-breath indirect calorimeters measure volumetric oxygen consumption (VO_2_) and carbon dioxide production (VCO_2_) at 21–85% FiO_2_ reliably but with bias at 85% FiO_2_ [25]. We have previously shown that the E-COVX metabolic module connected to a CARESCAPE™ R860 ventilator could reliably record spirometry and metabolic indices as early as 5 min after suctioning using different ventilatory modes in sedated, mechanically ventilated children [26,27].

There are few external cross-validation studies of predictive energy expenditure (eREE) equations in critically ill children or adolescents [23,28,29]. The aim of the present study was to externally cross-validate simplified predictive equations in critically ill children, using online continuous REE_IC_ measurements through indirect calorimetry. A secondary objective was to identify anthropometric, nutritional, or clinical factors that might influence REE_IC_, further affecting the accuracy of predictive equations in the acute phase of illness or injury.

## 2. Materials and Methods

### 2.1. Study Design

Critically ill children admitted to the academic Pediatric Intensive Care Unit (PICU) at the University Hospital, School of Medicine, University of Crete, Heraklion, from September 2014 through September 2018, and mechanically ventilated for ≥3 days were potential candidates to be enrolled in the study. The Ethics Committee of the Institutional Review Board approved the study (approval ID14494/2011/9-1-2012). All data were de-identified, and parents or guardians gave informed written consent. The study was conducted in accordance with the 1975 Declaration of Helsinki, as revised in 2013, following the International Conference on Harmonization (ICH)/Good Clinical Practice (GCP) standards [30].

In reporting this study, we used the STROBE Statement−Checklist for cross-sectional studies. Inclusion criteria: Hemodynamically stable, adequately sedated (Ramsey > 3), mechanically ventilated patients, with a Fractional Inspired Oxygen (FiO2) < 60%, a respiratory rate below 35 breaths-per-minute, and an endotracheal tube (ET) leak below 10% (inspiratory tidal volume (TVi) − expiratory tidal volume (TVe)/inspiratory TV × 100) were eligible for the study. Exclusion criteria: (1) Patients expected to be extubated within 48 h of admission; (2) on renal replacement therapy; (3) metabolic or endocrine disorders; (4) use of drugs known to affect energy expenditure, such as levothyroxine; (5) respiratory quotient (RQ) < 0.67 or > 1.3; and (6) unexpected interruption of the measurement (destabilization, need for intervention in the ventilation settings, or other).

### 2.2. Clinical Data

At the time of each metabolic measurement, the admission diagnosis, temperature, blood pressure, heart rate, sedation level by Ramsey scale, and main sedatives and vasoactive agents or inotropes were recorded. The last recorded temperature on a patient’s vital signs flowchart just before the REE_IC_ measurement was documented. The severity of illness was assessed using the PRISM-III and the PELOD-2 scores [31], and the amount of care was assessed using the Therapeutic Intervention Scoring System (TISS) [32]. The ventilatory settings at the time of the measurement and the route of nutrition support, and the total calories received for the 24-h period before metabolic measurement were also recorded. Energy intake was calculated from recorded intakes of enteral or parenteral nutrition and glucose-containing maintenance fluids. Underfeeding and overfeeding were defined according to the European Society for Clinical Nutrition and Metabolism (ESPEN) guidelines as intakes of <70% or >110% of REE_IC_, respectively [33].

### 2.3. Anthropometry

The following anthropometric parameters were identified: age, sex, actual weight, ideal weight, height, and body mass index (BMI). Weight was measured using calibrated electronic bed scales. Ideal weight was defined as the weight for the 50th percentile of the actual height of each patient. BMI was calculated as kg/m^2^. Standard deviations scores, known as z-scores, of weight, height, and BMI for sex and age were calculated using WHO and CDC calculators [34]. Malnutrition indices were derived from the BMI for age and sex z scores obtained at admission. Underweight was defined as BMI for sex and age z-score < −1.644, normal weight as −1.644 ≤ BMI z-score < 1.036, overweight as 1.036 ≤ BMI z-score < 1.644, and obesity as BMI z-score ≥ 1.644.

### 2.4. Indirect Calorimetry

An integrated gas exchange module (E-COVX) into the ventilator (Carescape R860; GE Healthcare, Milwaukee, WI, USA) was used to measure REE_IC_ through indirect calorimetry on PICU day 3 or 4. This module is able to reliably record spirometry and metabolic indices as early as 5 min after suctioning at different modes of ventilation [26,27]. It has no mixing chamber and sampling takes place with every breath. It has a fast differential paramagnetic O_2_ and infrared CO_2_ analyzer and a pneumotachograph housed in a connector, which measures inspired and expired volumes. In the P-Lite (15–300 mL) or D-Lite (>300 mL) flow sensor, located proximate to the Y-piece to the patients’ ET tube the flow measurement is based on the pressure drop across a special proprietary turbulent flow restrictor. It uses mathematical integration of flow and time-synchronized continuous gas sampling to provide data. The gas sample is continuously drawn from the connector to the gas analyzer unit of the module. Both O_2_ and CO_2_ measures are based on the side-stream principle. The E-COVX relies on tidal volume measurement for VO_2_ calculation. The pneumotachograph derives the tidal volume from the pressure difference across a fixed orifice, potentially influenced therefore by acute changes of resistance in the spirometry tubing and undetected leaks in the system. We consistently used a heat- and moisture-exchange filter alone, avoiding heated water bath humidification, followed by regular checks on the spirometry tubing and checks for tidal volume consistency between the module and the ventilator.

Measurements were made between 9 am and 12 pm when there had been a minimum of 45 min with no major physical activity, such as physiotherapy or dressing change. After an initial 10-min stabilization period, REE_IC_ was measured for 30 min, during which time there was no interference with the child. The module uses the modified Weir formula (REE_IC_ (kcal/day) = [3.941 × VO_2_ + 1.106 × VCO_2_] × 1440 and displays a 5-min average for REE_IC_ but can display the 1-min averages with the S/5 Collect 1.0 software (Datex-Ohmeda, GE Healthcare, USA). Steady state was defined as a period of at least 5 min with less than 10% fluctuation in VO_2_ and VCO_2_, and less than 5% fluctuation in respiratory quotient (RQ), which is the ratio of VCO_2_: VO_2_. Measurements with RQ outside the physiologic range (>1.3 or <0.67) were excluded.

### 2.5. Prediction Equations

To avoid unpredictable anthropometric alterations and for logistic reasons, predictive equations were estimated at the same time in the morning, between 9 am–12 pm. The eREE equations for each patient were calculated using actual (or ideal in obese patients) weight using the following equations (Table S1, Supplementary Materials): Harris–Benedict [2], Schofield H-W (WHO) [5], FAO/WHO)/UNU [3], Henry (Oxford) [7], IOM [4], Lawrence (Equation (3)) [7], Kaneko [8], Dietz [9], Maffeis [10], Molnár [11], Muller [12], Mifflin [15], Caldwell–Kennedy [18], Lazzer (Equation (1)) [13], and the PICU-specific White (Equation (2)) [19] and Meyer (Equation (C)) [20]. The accuracy of these equations was defined as prediction values that fell within 90% to 110% of the measured REE_IC_ (±10% accuracy). All other predictions falling outside this range were deemed inaccurate. As a control equation, the age-specific recommended dietary allowances (RDA) for healthy children were simultaneously calculated [21].

Basal metabolism was calculated based on the Schofield equation. The metabolic state for each patient was determined using the ratio of measured REE_IC_ to eREE based on the Schofield equation, as has been previously suggested [35,36,37]. Patients were classified in the following metabolic patterns: normometabolic when REE_IC_/eREE_Schofield_ = 90–110%, hypometabolic when REE_IC_/eREE_Schofield_ < 90%, and hypermetabolic when REE_IC_/eREE_Schofiled_ > 110%.

### 2.6. Statistical Analysis

The normality of the distribution was assessed using the Shapiro–Wilk test. Descriptive data are reported as means and standard deviation (SD) or median and interquartile range (IQR) in case of skewed distributions, or as frequencies and percentages when appropriate. The accuracy of the eREE compared to REE_IC_ measured by indirect calorimetry was assessed through the calculation of bias and precision. Bias was defined as the mean difference between the measurements obtained from the eREE and REE_IC_. Precision was defined by the 95% limits of the agreement including both systematic (bias) and random error. The percentage of predicted values of an equation within 10% of REE_IC_ was considered a measure of accuracy on a cohort or sub-cohort level. The relative variability (dispersion) and repeatability were assessed by calculating the coefficient of variation (CV) which is the ratio of the standard deviation to the mean of the population. The reliability was assessed by the intraclass correlation coefficient (ICC), calculated using the two-way mixed (Cronbach’s alpha). ICC was interpreted as follows: below 0.50: poor; between 0.50 and 0.75: moderate; between 0.75 and 0.90: good; above 0.90: excellent [38]. A linear regression model (backward method) was adopted to examine whether any of the recorded anthropometric, clinical, and nutritional variables are independently associated with REE_IC_. We first used univariate models to test if any of the studied variables were related to REE_IC_, with just one explanatory variable at a time; afterward, all variables that had shown a relaxed *p*-value of less than or equal to 0.1 were included in the multivariable models. A two-sided significance level of 0.05 was used for statistical inference. Statistical analysis software (version 28; SPSS, Chicago, IL, USA) was used for all analyses and GraphPad Prism 9.0 (GraphPad Software, Inc., San Diego, CA, USA) was used for the Bland–Altman analyses and illustrations.

## 3. Results

### 3.1. Study Population

During the study period, 735 patients were admitted to the PICU, of which 176 were eligible for inclusion. However, 23 patients were not enrolled due to logistical reasons (*n* = 12), technical reasons (*n* = 7), or no informed consent (*n* = 4). Demographic, anthropometric, clinical, and metabolic characteristics are shown in Table 1.

Less than half of the patients (*n* = 69; 45.1%) had a BMI within the normal range for their sex and age. Thirty (19.6%) patients were underweight (19.6%), and fifty-four were overweight (10.5%) or obese (24.8%). All patients received sedation and analgesia. Mortality was 3.9%. Nutritional support was provided enterally (90.9%) or parenterally (9.1%). Patients’ feeding status on PICU day three revealed that two-thirds of the patients were either underfed (39.8%) or overfed (27.6%). Normal weight patients received targeted nutrition in only 36.7%, while 36.7% were underfed and 26.7% were overfed. In contrast, 55.6% of obese patients were underfed whereas 36% of underweight patients were overfed (Table 1).

### 3.2. Performance of Predictive Equations

The calculated eREE either underestimated (median 606, IQR 512; 784 kcal/day) or overestimated (1126.6, 929; 1340 kcal/day) REE_IC_ compared with indirect calorimetry (928.3, 651; 1239 kcal/day). Comparison analysis between resting energy expenditure measured by indirect calorimetry and calculations through individual predictive equations are presented in Table 2.

**Figure 1:**
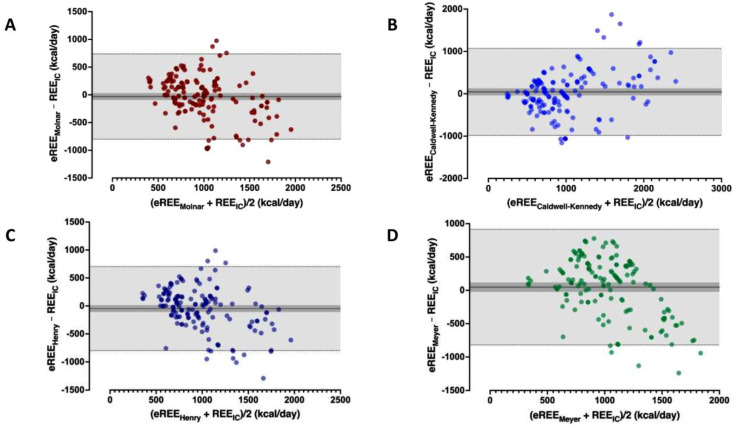
Bland–Altman plot whereby estimated by predicted equations’ resting energy expenditure (eREE) is compared to REE measured by IC (REE_IC_) at ICU Day-3 or 4. (**A**). Molnar eREE compared to REE_IC_. (**B**). Caldwell–Kennedy eREE compared to REE_IC_. (**C**). Henry (Oxford) eREE compared to REE_IC_. (**D**). Meyer equation-C eREE compared to REE_IC_. The solid line indicates the percentage of agreement bias (%) and the light shade with the fine dotted lines indicates the limits of agreement (bias ± (1.96 × SD) = precision). Dark shade represents the 95% confidence intervals of the mean (bias).

The calculated age and sex-specific RDA, grossly overestimated REE_IC_ )median 1320 (IQR 880; 2365) kcal/day). These differences resulted in significant biases of −342 to 592 kcal (95% limits of agreement (precision) −1107 to 1380 kcal/day). Even predictive equations with small bias (Molnar, Caldwell–Kennedy, Henry (Oxford), Meyer) exhibited extended dispersion of values as visualized by the 95% limits of agreement in the Bland–Altman plots (Figure 1). Compared to indirect calorimetry, old or new equations, irrelevant to the established age, nutrition, race, or illness-related status, presented a large bias and small precision, indicated by the wide 95% limits of agreement in the Bland–Altman plots (Figures S1–S3).

Paired eREE–REE_IC_ differences were significant for most predictive equations (Wilcoxon matched-pairs signed rank test, medians of differences −282 to +627, *p* < 0.002) except for the Molnar, Caldwell–Kennedy, Henry (Oxford), Meyer equations (−8 to +137, *p* > 0.05). These equations, however, were also inaccurate, presenting a wide dispersion of values as expressed by a high coefficient of variation (809–1242%), in accordance with their high bias and limits of agreement (Table 2).

The equations’ reliability, as assessed by the ICC, although significant (*p* < 0.001), varied at moderate levels between 0.51 and 0.74 and was consistent across sub cohorts of obese, overweight, and underweight patients (Cronbach’s alpha, Table 3).

**Figure 2:**
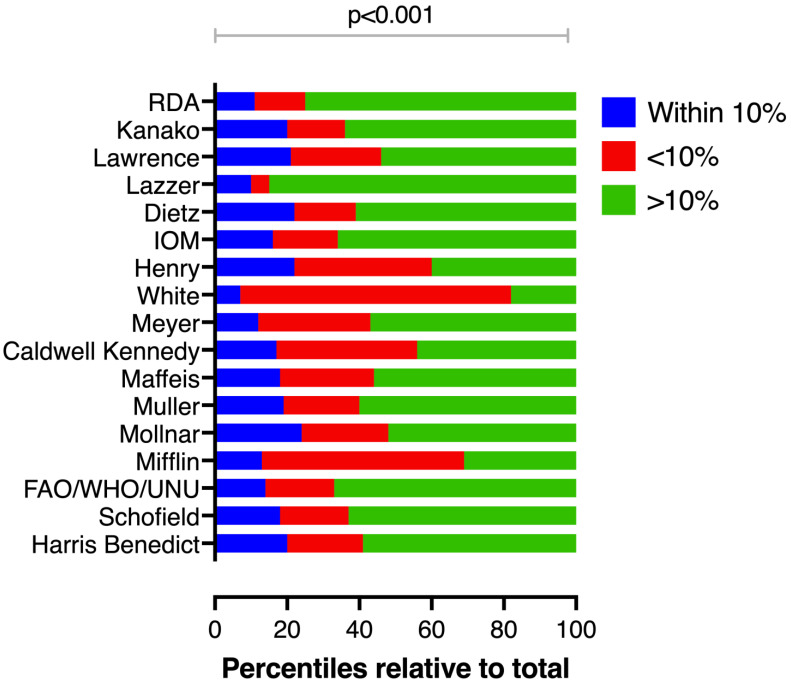
Percentages of estimated resting energy expenditure (eREE) values of an equation within 10% of resting energy expenditure measured by IC (REE_IC_) (blue color). Inaccuracy profiles varied from underestimation (red color) to overestimation (green color) of the patient’s energy needs. Clinically significant percentage error (eREE − REE_IC_)/ REE_IC_ (%) was considered a difference of ≥ ±10% and it was significantly lower than an expected minimum accuracy of 50%.

Despite the moderate reliability, the 10% accuracy rate ranged from 6.5% to a maximum of 24.2%, and it was significantly lower than an expected minimum accuracy overall and across nutrition status sub-cohorts (Table 3). Inaccuracy profile varied from underestimation (White, median −31%, IQR −44%; −9.5%) to overestimation (Lazzer, median 71.5%, IQR 28.6%; 138%) of the patient’s energy needs (*p* < 0.001) (Figure 2).

### 3.3. Malnutrition and Factors Independently Associated with REE_IC_

**Figure 3:**
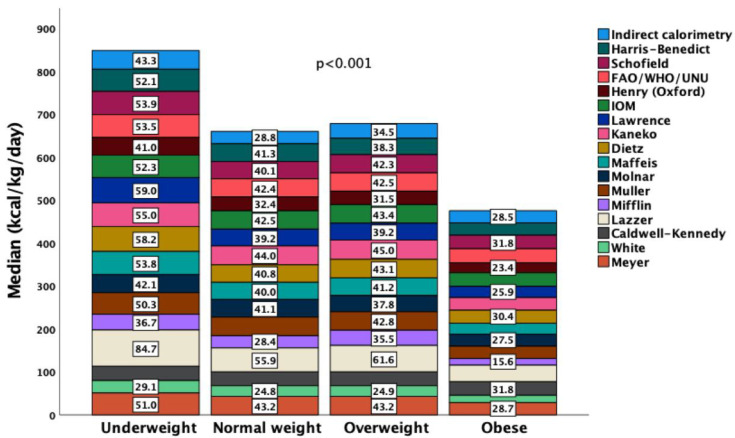
Measured by indirect calorimetry resting energy expenditure (REE_IC_) (kcal/kg/day) was higher in the underweight and lower in the obese compared to other sub-cohorts (*p* = 0.017). All predicted equations also calculated higher kcal/kg/day in the underweight compared to overweight and obese patients (*p* < 0.001). Numbers in the white boxes indicate the medians of the equations.

Measured by indirect calorimetry, REE_IC_ (kcal/kg/day) was higher in the underweight and lower in the obese compared to other sub-cohorts (*p* = 0.017). All predicted equations also calculated higher kcal/kg/day in the underweight compared to overweight and obese patients (*p* < 0.001) (Figure 3).

**Figure 4:**
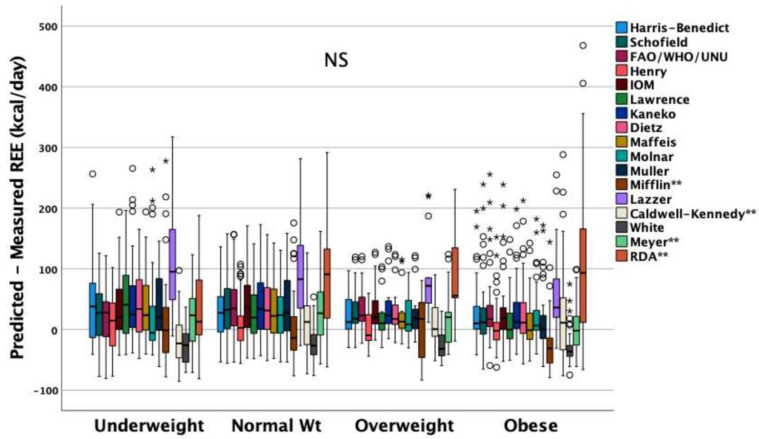
Paired estimated by predicted equations’ resting energy expenditure (eREE) and REE measured by IC (REE_IC_) differences did not differ among malnutrition groups for most predicted equations, apart from the Mifflin (*p* = 0.016), Caldwell–Kennedy (*p* = 0.039), Meyer (*p* = 0.042), and RDA (*p* < 0.01) equations. The bold black line in box plots indicates the median per group, the bottom of the box indicates the 25^th^ percentile and the top of the box represents the 75^th^ percentile; the T-bars (whiskers) and horizontal lines show minimum and maximum values of the calculated non-outlier values; circles are the outliers, asterisks are the extreme outliers.

Paired eREE-REE_IC_ differences did not differ among malnutrition groups for most predicted equations, apart from the Mifflin (*p* = 0.016), Caldwell–Kennedy (*p* = 0.039), Meyer (*p* = 0.042), and RDA (*p* < 0.01) equations (Figure 4).

In a linear regression model (stepwise, backward method), only a younger age (Beta −0.49, *p* < 0.001) was independently associated with the measured REE_IC_. None of the BMI nutrition status (overweight, obesity), the severity of illness (PRISM, TISS, PELOD), diagnostic category, outcome, temperature, heart rate, lactate, vasoactive drugs, neuromuscular blockade, or energy intake were independently associated with the REE_IC_.

## 4. Discussion

The accurate determination of energy needs in critically ill children is vital because underfeeding and overfeeding are both associated with undesirable consequences. Although IC is considered the gold standard for assessing REE in ICU patients, several predictive equations, developed from measured energy expenditure based on various numbers of healthy non-hospitalized subjects, are commonly used in clinical practice. In this study, we evaluated commonly used previously validated equations and found that even the most accurate equations had an unacceptably high error. We showed that recommended or not, PICU-related or not, the older or newly predictive equations presented large biases and small precisions, as indicated by the wide 95% limits of agreement in the Bland–Altman plots, significant paired differences, and high coefficients of variation. We also showed that although sixteen predicted equations exhibited moderate reliability, the clinically acceptable 10% accuracy rate ranged from only 6.5% to a maximum of 24.2%, with the inaccuracy varying from −31% to +71.5% of the measured patients’ energy needs. Finally, we demonstrated that, apart from a younger age, malnutrition, clinical characteristics, temperature, vasoactive drugs, neuromuscular blockade, and energy intake did not affect REE_IC_ and thereby eREE.

A novel finding of this study is that the inaccuracy of the assessed predictive equations did not correlate with the established time (old or new), age range (pediatric, adult), malnutrition status, race, illness-related status (healthy, PICU), or recommendation by scientific societies (Schofield, WHO, IOM). For predicting energy requirements, the Schofield [5] and FAO/WHO/UNU [3] equations have been previously recommended for the healthy pediatric population [39], while in a population with obesity, the Molnár [11] and Dietz [9] equations performed most accurately. For patients receiving mechanical ventilator support, the Harris–Benedict predicted more accurately than other equations, but with a wide error range (±500 kcal) [17]. In our critically ill, mechanically ventilated patients, predicted equations either underestimated or overestimated REE, compared with measured REE_IC_. All predictions presented significant matched paired eREE-REE_IC_ differences, a wide dispersion of values as expressed by high coefficients of variation, significant biases of −342 to 592 kcal, and poor precision (−1107 to 1380 kcal/day). Most of the equations overestimated REE_IC_, erroneously calculating higher energy needs of critically ill patients. Findings of previous studies using indirect calorimetry support our conclusion that children do not become hypermetabolic during critical illness [36] and that improved PICU-specific prediction methods are still imprecise in critically ill children [23,40,41,42,43].

Our data suggest that simple predictive equations may lead to overfeeding in critically ill children and less often to underfeeding. A U-shaped association between mortality and energy intake revealed the importance of personalized energy support and the need to prevent overfeeding and underfeeding [44]. Two recent meta-analyses showed a reduction in ICU mortality when feeding protocols were based on REE_IC_ [45] compared to eREE [46]. Nutrition guidelines recommend measuring REE using a validated indirect calorimeter to guide nutritional support in critically ill infants and children after the acute phase [47]. Alternatively, the Schofield equation is recommended to estimate REE [47], which we showed to be one of the most inaccurate. Imprecise predictive equations that overestimated REE_IC_ more than others were the RDA (95% limits of agreement −1101 to 2585 kcal/day), Lazzer (−196 to 1380 kcal/day), IOM (−593; 1011 kcal/day), Kaneko (−549 to 967 kcal/day), Schofield H-W (−652 to 1021 kcal/day), and Dietz-(598 to 959 kcal/day) equations. Although the FAO/WHO/UNU, Harris–Benedict, Maffeis, Lawrence, and Muller equations’ overestimation bias was smaller, they were inaccurate with wide 95% limits of agreement. Finally, the two equations that mostly underestimated REE_IC_ were the White (−1107 to 422 kcal/day) and Mifflin (−966.8 to 575 kcal/day) equations.

In accordance with the results of the Vazquez Martinez study in the early postinjury period [17], we found the Caldwell–Kennedy equation to be among the four less inaccurate predictors of energy expenditure in ventilated, critically ill children. However, even the four predictive equations with the smallest bias, Molnar (−32 kcal/day), Caldwell–Kennedy (44 kcal/day), Henry (Oxford) (−47 kcal/day), and Meyer (47 kcal/day), exhibited extended dispersion of values as visualized by a high coefficient of variation (809-1242) and wide limits of agreement (+539.55; 1378.03 kcal/day). In the absence of IC, American Society for Parenteral and Enteral Nutrition (ASPEN) guidelines suggested that a published predictive equation or a simplistic weight-based equation (25–30 kcal/kg/d) be used in adults to determine energy requirements [48]. However, if predictive equations are used to estimate the energy need, hypocaloric nutrition (below 70% of eREE) should be preferred over isocaloric nutrition for the first week of ICU stay as per ESPEN guidelines [33].

In our series, more than half of the patients were malnourished, whereas two-thirds were underfed or overfed. In addition, both indirect calorimetry and predicted equations calculated higher kcal/kg in the underweight compared to overweight and obese patients. Following the same trend, obese patients were underfed (70.4%), whereas 36% of underweight patients were overfed. It has been suggested that patients who are at high nutrition risk or severely malnourished should be advanced to provide >80% of REE_IC_ or eREE and protein within 48–72 h to achieve the clinical benefit of early enteral nutrition while monitoring for refeeding syndrome [48]. Hypocaloric parenteral nutrition dosing (80% of eREE) with adequate protein (≥1.2 g protein/kg/d) should also be considered in high-risk or severely malnourished patients requiring parenteral nutrition over the first week in ICU [48]. Regarding obesity, the guidelines suggest that the goal of enteral nutrition should not exceed 65%–70% of the target REE_IC_ [48]. Personalized nutritional adjustments may impact PICU length of stay, readmission rates, quality of life [49], and long-term rehabilitation success [50]. Scientific societies recommend measuring REE by IC in malnourished children and/or suspected altered metabolism. According to these criteria, more than 70% of PICU patients are candidates for IC measurement [51]. Our finding that <25% of the equations predicted REE_IC_ within ±10% of the indirect calorimetry REE_IC_ exaggerates the results of a systematic review study, showing that no equation predicted REE_IC_ within ±10% in >50% of observations [52].

Most of our patients were hypometabolic, in accordance with previously published data (5, 6, 15–17). Several factors have been implicated to explain the hypometabolism of critically ill children, such as coma, mechanical ventilation, analgesia, sedation, neuromuscular blockade, and malnutrition. It is the first time, however, to demonstrate that none of the malnutrition status, the severity of illness, diagnostic category, outcome, temperature, heart rate, lactate, vasoactive drugs, neuromuscular blockade, or energy intake were independently associated with the REE_IC_ inaccuracy. In agreement with findings of an adult study in critically ill medical patients [53], we showed that only a younger age is independently associated with indirect calorimetry measurements in mechanically ventilated children. Accordingly, except for age, none of the estimated nutritional or clinical confounders might indirectly affect the REE_IC_-eREE difference. This hypothesis is further supported by the fact that PICU-related equations did not perform better than other predictive equations.

One of the limitations of this study is the small sample size, although it is in the upper range of similar studies, including sixteen predictive equations, older, recent, adult, pediatric, PICU-related, and nutrition status-related equations. In addition, the timing of the IC measurements in this prospective cross-sectional study only reflects the acute and not the recovery metabolic phase of illness. According to the ESPEN guidelines, every critically ill patient staying for more than 48 h in the ICU should be considered at risk for malnutrition [33]. We measured REE on ICU Day 3 or 4 since it has been previously shown that non-inhibitable endogenous energy is produced in the acute phase of critical illness due to a catabolic state [50]. Since the non-measurable, adapted to acute illness endogenous effect dissipates by Day 4 [54], it is recommended to commence early enteral nutrition within 24 h of admission [55], and to increase it in a stepwise fashion until the goal for delivery is achieved using a feeding protocol [47], to avoid overfeeding and mitochondrial exhaustion by targeting REE_IC_ during the acute stress period [49,56]. Adult guidelines also recommend that hypocaloric nutrition (not exceeding 70% of REE_IC_) should be administered in the early phase of acute illness and that isocaloric nutrition should be progressively implemented after the early phase of acute illness [33]. Because of the unpredictable effects of a critical illness on metabolism, the considerable variation in REE, and the progressive hypermetabolism, IC should be used daily in assessing nutrition in ICU patients [57]. After the acute phase, energy intake should account for energy deficits, physical activity, or exercise, and growth [47]. Recently developed self-calibrating and simple-to-operate instruments, with implemented artificial intelligence, have built-in algorithms for the detection and deletion of aberrant periods of measurements resulting from breathing variability [58]. Future developments of metabolic cart technology to reliably monitor REE_IC_ continuously in states of respiratory and circulatory instability, using various ventilatory settings, including non-invasive ventilation, are expected to facilitate the daily application of IC in an intensive care setting.

## 5. Conclusions

All available prediction equations for calculating energy needs are inaccurate for individual patients, either underestimating or overestimating REE compared with indirect calorimetry. Apart from a younger age, malnutrition, clinical characteristics, temperature, vasoactive drugs, neuromuscular blockade, and energy intake did not affect REE_IC_ and thereby the accuracy of the predictive equations. Sixteen predictive equations may result in under- or overfeeding and cannot substitute for indirect calorimetry measurement of energy expenditure in guiding the personalization of nutrition delivery in pediatric intensive care patients.

## Figures and Tables

**Table 1:** Demographic and clinical characteristics.

*N* = 153					
Demographic		Clinical Data		Indirect Calorimetry	
Age (years)	7.5 (5; 12.5)	PRISM score	9 (6; 15)	REE (kcal/day)	928 (651; 1238)
Sex (male/female)	108/45, (70.6%/29.4%)	TISS score	41 (36; 46)	REE (kcal/kg/day)	32.3 (23.0; 48.3)
Anthropometric		PELOD score	7 (2; 18)	VO_2_ (mL/min)	134 (95.5; 176.8)
Body weight (kg)	25 (16.5; 41.5)	FiO_2_ (%)	35 (30; 50)	VCO_2_ (mL/min)	111 (74.6; 153.2)
Height (cm)	130 (111; 148)	pH	7.39 (7.35; 7.43)	Respiratory Quotient	0.85 (0.77; 0.91)
BMI (kg/m^2^)	16.6 (15.2; 20.6)	pO_2_ (mmHg)	96 (87; 111)	Metabolic state * (kcal/day)	88.5 (69.7; 106.7)
z-score weight for age	0.42 (−1.2; 1.2)	pCO_2_ (mmHg)	36 (33.5; 39.1)	Metabolic pattern **	
z-score height for age	−0.03 (−0.54; 0.55)	HCO_3_ (mEq/L)	22.3 (19.6; 24.5)	Normometabolic	42 (27.5%)
z-score BMI for age	0.47 (−0.98; 1.65)	Heart Rate (bpm)	100 (80: 119)	Hypometabolic	82 (53.6%)
BMI nutrition status		Respiratory rate (bpm)	22 (18; 25.8)	Hypermetabolic	29 (19%)
Underweight	30 (19.6%)	Systolic Blood Pressure (mmHg)	97 (78; 107)	Nutrition day 3	
Normal BMI	69 (45.1%)	Body Temperature (°C)	37.2 (36.8; 37.8)	Energy intake (kcal/day)	720 (480; 1000)
Overweight	16 (10.5%)	Neuromuscular blockade, yes	11/66 (16.7%)	Energy intake (kcal/kg/day)	27.4 (16; 41.7)
Obese	38 (24.8%)	Vasoactive, yes	40/82 (56.3%)	Energy intake/REE ratio	
Clinical diagnosis		Lactate (mg/dL)	10.8 (6.3; 18)	Energy intake/REE (%)	88.2 (47.7; 112.9)
Respiratory failure	40 (26.2%)	Glucose (mg/dL)	103 (93; 121)	Feeding status	
Sepsis	27 (17.6%)	Albumin (mg/dL)	3.1 (2.7; 3.4)	Adequate	40/123 (32.5%)
Surgical	11 (7.2%)	C-Reactive Protein (mg/dL)	8 (1.3; 16)	Underfeeding	49/123 (39.8%)
Organ failure	4 (2.6%)	Length of Stay (days)	14 (6.5; 23.5)	Overfeeding	34/123 (27.6%)
Trauma	41 (26.8%)	Mechanical Ventilation (days)	12 (5; 18)	Underfeeding/Obese	15/27 (55.6%)
Neurologic	30 (19.6%)	Mortality	6 (3.9%)	Overfeeding/Underweight	9/25 (36%)

Continuous variables are reported as 50th (median) and 25th and 75th percentiles (interquartile range, within brackets). Discrete variables are reported as the number and proportion (within brackets) of subjects with the characteristic of interest. BMI = Body Mass Index; PRISM, Pediatric Risk of Mortality; TISS = Therapeutic Intervention Scoring System; PELOD = Pediatric Logistic Organ Dysfunction; REE = Resting Energy Expenditure; VO_2_ = Volumetric Oxygen Consumption; VCO_2_ = Volumetric Carbon Dioxide Production. * Metabolic state = ratio of measured REE_IC_ to eREE based on the Schofield equation. ** Normometabolic REE_IC_/eREE_Schofield_ = 90–110%; hypometabolic REE_IC_/eREE_Schofield_ < 90%; hypermetabolic when REE_IC_/eREE_Schofield_ > 110%.

**Table 2:** Comparison analysis between resting energy expenditure measured by indirect calorimetry and calculations through predictive equations (kcal/day).

	REE (kcal/Day)			Agreement-Precision *			Paired Differences-Variability ^#^			
Compared Equation	IQR 25th	Median	IQR 75th	Mean Bias	SD	Limits of Agreement	Medan of Differences	IQR 25th; 75th	CV (%)	*p* Value
*n* = 153										
Indirect Calorimetry	651.35	928.30	1238.39							
Harris–Benedict	920.17	1083.41	1263.46	142	391	−624; 908	174	−48; 388	275	<0.001
Schofield H-W	864.58	1057.30	1439.47	185	427	−652; 1021	191	−41; 469	231	<0.001
FAO/WHO/UNU	727.13	935.25	1216.50	146	398	−634; 926	142	−32; 430	273	<0.001
Henry (Oxford)	739.37	860.654	1172.30	−47	383	−798; 703	5	−236; 176	809	0.421
IOM	937.55	1090.30	1404.64	209	409	−593; 1011	205	21; 481	196	<0.001
Lawrence	885.64	995.93	1296.82	81	384	−672; 834	130	−119; 342	475	<0.002
Kaneko	1016.62	1122.27	1357.78	209	387	−549; 967	211	21; 468	185	<0.001
Dietz	919.61	1072.04	1393.22	181	397	−598; 959	219	−25; 434	220	<0.001
Maffeis	921.37	1048.95	1215.10	87	388	−673; 846	127	−134; 396	448	<0.002
Molnar	929.43	1126.62	1339.89	−32	393	−802; 739	−8	−207; 235	1242	0.843
Muller	869.15	1062.50	1471.10	96	393	−674; 866	111	−120; 352	410	<0.001
Mifflin	561.30	769.90	1050.96	−159.9	393.3	−966.8; 575	−926	−1235; −650	201	<0.001
Lazzer (equation 1)	1346.00	1548.00	1831.00	592	402	−196; 1380	627	385; 869	68	<0.001
Caldwell–Kennedy	539.55	806.37	1378.03	44	524	−983; 1071	35	−213; 291	1187	0.358
White (equation 2)	512.07	606.06	784.21	−342	390	−1107; 422	−282	−520; −69	114	<0.001
Meyer (equation C)	800.56	1054.00	1302.86	47	442	−820; 915	137	−264; 382	935	0.058
RDA	880.00	1320.00	2365.00	742	940	−1101; 2585	568	58; 1210	127	<0.001

Continuous variables are reported as median (interquartile range). Abbreviations: IQR = interquartile range; SD = Standard Deviation; CV = Coefficient of Variation; REE = Resting Energy Expenditure.* Bland–Altman; ^#^ Wilcoxon matched pairs signed rank test. Statistical significance was considered for *p* < 0.05.

**Table 3:** Reliability by intraclass correlation coefficient (average measures) and 10% accuracy of the studied equations of predicted energy expenditure in comparison to the resting energy expenditure measured by indirect calorimetry.

	Reliability ^		Accuracy ^#^											
Compared Equation	All *n* = 153						Underweight *n* = 30		Normal Weight *n* = 69		Overweight *n* = 16		Obese *n* = 38	
	ICC (Average Measures)	*p* Value	Within ±10%	<−10%	>+10%	*p* Value *	Within ±10%	*p* Value*	Within ±10%	*p* Value *	Within ±10%	*p* Value *	Within ±10%	*p* Value *
Harris–Benedict	0.699 (0.58; 0.78)	<0.001	20.3	20.9	58.8	<0.001	2/28	<0.001	13/56	<0.001	5/11	0.134	11/27	<0.01
Schofield	0.73 (0.63; 0.80)	<0.001	17.6	19	63.4	<0.001	5/25	<0.001	13/56	<0.001	2/14	0.003	8/30	<0.001
FAO/WHO/UNU	0.74 (0.64; 0.81)	<0.001	14.4	19	66.7	<0.001	3/27	<0.001	12/57	<0.001	1/15	<0.001	7/31	<0.001
Henry (Oxford)	0.70 (0.59; 0.78)	<0.001	21.6	37.9	40.5	0.008	3/27	<0.001	17/52	<0.001	2/14	0.003	12/26	0.023
IOM	0.72 (0.61; 0.79)	<0.001	15.7	17.6	66.7	<0.001	4/26	<0.001	12/57	<0.001	3/13	0.012	7/31	<0.001
Lawrence	0.65 (0.52; 0.75)	<0.001	20.9	24.8	54.2	<0.001	6/24	<0.001	12/57	<0.001	5/11	0.134	9/329	<0.001
Kaneko	0.67 (0.55; 0.76)	<0.001	20.3	15.7	64.1	<0.001	6/24	<0.001	10/59	<0.001	3/13	0.012	12/26	0.023
Dietz	0.72 (0.61; 0.79)	<0.001	21.6	17	61.4	<0.001	5/25	<0.001	10/59	<0.001	5/11	0.134	11/27	0.009
Maffeis	0.62 (0.47; 0.72)	<0.001	17.6	26.1	56.2	<0.001	5/25	<0.001	12/57	<0.001	4/12	0.046	6/32	<0.001
Molnar	0.68 (0.56; 0.77)	<0.001	24.2	23.5	52.3	<0.001	6/24	<0.001	14/55	<0.001	5/11	0.134	12/26	0.023
Muller	0.67 (0.55; 0.76)	<0.001	19	20.9	60.1	<0.001	3/27	<0.001	11/58	<0.001	3/13	0.012	12/26	0.023
Mifflin	0.68 (0.57; 0.77)	<0.001	13.1	55.6	31.4	<0.001	5/25	<0.001	10/59	<0.001	2/14	0.003	3/35	<0.001
Lazzer (equation 1)	0.69 (0.58; 0.77)	<0.001	9.8	4.6	85.6	<0.001	4/26	<0.001	7/62	<0.001	0/16	-	4/34	<0.001
Caldwell–Kennedy	0.72 (0.61; 0.79)	<0.001	17	38.6	44.4	<0.001	7/23	<0.001	7/62	<0.001	5/11	0.003	7/31	<0.001
White (equation 2)	0.60 (0.46; 0.71)	<0.001	6.5	75.2	18.3	<0.001	2/28	<0.001	4/65	<0.001	1/15	<0.001	3/35	<0.001
Meyer (equation C)	0.51 (0.32; 0.64)	<0.001	12.4	30.7	56.9	<0.001	2/28	<0.001	11/58	<0.001	0/16	-	6/32	<0.001
RDA	0.58 (0.42; 0.69)	<0.001	10.5	14.4	75.2	<0.001	8/22	0.011	4/65	<0.001	1/15	<0.001	3/35	<0.001

Continuous variables are reported as median (interquartile range). Abbreviations: RDA = Recommended Dietary Allowances; ICC = Intraclass Correlation Coefficient. ^ Reliability by the Intraclass Correlation Coefficient using the two-way mixed consistency (average ICC measures identical to Cronbach’s Alpha values); ^#^ Clinically significant percentage error (REE_VCO2_ − REE_IC_)/REE_IC_ (%); * Nonparametric x^2^ test; Statistical significance was considered for *p* < 0.05.

## Data Availability

The datasets generated and analyzed during the current study are not publicly available because the database is very extensive and includes data from other studies complementary to this, but are available from the corresponding authors upon reasonable request.

## Supplementary Materials

The following supporting information can be downloaded at: https://www.mdpi.com/article/10.3390/nu14194149/s1, Table S1: Predicted energy expenditure equations compared to indirect calorimetry for calculating energy expenditure in critically ill children; Figure S1: Bland–Altman plot whereby estimated by predicted equations’ resting energy expenditure (eREE) is compared to REE measured by IC (REE_IC_) at ICU Day-3 or 4. A. Harris– Benedict eREE compared to REE_IC_. B. Schofield (height and weight, WHO) eREE compared to REE_IC_. C. Mifflin eREE compared to REE_IC_. D. Muller eREE compared to REE_IC_. The solid line indicates the percentage of agreement bias (%) and the light shade with the fine dotted lines indicates the limits of agreement (bias ± (1.96 × SD) = precision). Dark shade represents the 95% confidence intervals of the mean (bias).; Figure S2: Bland–Altman plot whereby estimated by predicted equations’ resting energy expenditure (eREE) is compared to REE measured by IC (REE_IC_) at ICU Day-3 or 4. A. Maffeis eREE compared to REE_IC_. B. White (Equation (2)) eREE compared to REE_IC_. C. Institute for Medicine of the National Academies and Food and Nutrition Board (IOM) eREE compared to REE_IC_. D. Dietz eREE compared to REE_IC_. The solid line indicates the percentage of agreement bias (%) and the light shade with the fine dotted lines indicates the limits of agreement (bias ± (1.96 × SD) = precision). Dark shade represents the 95% confidence intervals of the mean (bias); Figure S3: Bland–Altman plot whereby estimated by predicted equations’ resting energy expenditure (eREE) is compared to REE measured by IC (REE_IC_) at ICU Day-3 or 4. A. FAO/WHO/UNU eREE compared to REE_IC_. B. Lazzer (Equation (1)) eREE compared to REE_IC_. C. Lawrence-3 eREE compared to REE_IC_. D. Kaneko eREE compared to REE_IC_. The solid line indicates the percentage of agreement bias (%) and the light shade with the fine dotted lines indicates the limits of agreement (bias ± (1.96 × SD) = precision). Dark shade represents the 95% confidence intervals of the mean (bias). References [2,3,4,5,6,7,8,9,10,11,12,13,15,17,18,19,20].
