# Supplementary material for: External Validation of Equations to Estimate Resting Energy Expenditure in Critically Ill Children and Adolescents with and without Malnutrition: A Cross-Sectional Study
Source: Nutrients. 2022 Oct 6;14(19):4149. doi: 10.3390/nu14194149 (PMC9572704; doi:10.3390/nu14194149)
Supplement: Supplementary file 1 [file nutrients-14-04149-s001.zip › Table S1.pdf]

Table S1. Predicted energy expenditure equations compared to indirect calorimetry for calculating energy expenditure in critically ill children.

**Reference**	**Clinical condition**<br>**Age range (years)**	**Predictive equation (kcal/day)**
**Harris-Benedict [1]**	Healthy; 21-70	
• Male		66.473 + (13.7516 x Weight) + (5.0033 x Height) - (6.755 x Age)
• Female		655.0955 + (9.5634 x Weight) + (1.8496 x Height) - (4.6756 x Age)
**Schofield H-W (WHO) [2]**	Healthy; 3-18	
<3 years old		
• Male		(0.167 x Weight) + (1517.4 x Height) - 617.6
• Female		(16.252 x Weight) + (1023.2 x Height) - 413.5
3-10 years old		
• Male		(19.6 x Weight) + (103.3 x Height) + 414.9
• Female		(16.97 x Weight) + (161.8 x Height) + 371.2
10-18 years old		
• Male		(16.25 x Weight) + (137.2 x Height) + 515.5
• Female		(8.365 x Weight) + (465 x Height) + 200
**FAO/WHO/UNU [3]**	Healthy; 3-18	
<3 years old		
• Male		(60.9 x Weight) - 54
• Female		(61 x Weight) - 51
3-10 years old		
• Male		(22.7 x Weight) + 495
• Female		(22.5 x Weight) + 499
10-18 years old		
• Male		(16.6 x Weight) + (77 x Height) + 572
• Female		(7.4 x Weight) + (482 x Height) + 217
**Henry (Oxford) W-H [4]**	Healthy; 3-18	
<3 years old		
• Male		(28.2 x Weight) + (859 x Height*) - 371
• Female		(30.4 x Weight) + (703 x Height*) - 287
3-10 years old		
• Male		(15.1 x Weight) + (74.2 x Height*) + 306
• Female		(15.9 x Weight) + (210 x Height*) + 349
10-18 years old		
• Male		(15.6 x Weight) + (266 x Height*) + 299
• Female		(9.40 x Weight) + (249 x Height*) + 462
**Institute for Medicine of the National Academies and Food and Nutrition Board (IOM) [5]**	Healthy; 0-18	
• Male		68 - (43.3 x Age) + (712 x Height) + (19.2 x Weight)
• Female		189 - (17.6 x Age) + (625 x Height) + (7.9 x Weight)
**Lawrence (Equation 3) [6]**	Healthy; 4-11	

		632.4 + (15.66 x Age) + (9.53 x Weight)
**Kaneko [7]**	Healthy; 6-17	
• Male		(14.4 x Weight) + (5.09 x Height) - (34 x Age) + 403
• Female		(7.64 x Weight) + (4.22 x Height) - (22.5 x Age) + 526
**Dietz [8]**	Healthy; 6-17	
• Male		(16.6 x Weight) + (77 x Height*) + 572
• Female		(7.4 x Weight) + (482 x Height*) + 217
**Maffeis [9]**	Healthy; obese; 6-10	
• Male		1.287 + (28.6 x Weight) + (23.6 x Height) - (69.1 x Age) #
• Female		1.552 + (35.8 x Weight) + (15.6 x Height) - (36.3 x Age) #
**Molnár [10]**	Healthy; obese; 10-16	
• Male		(50 x Weight) + (25.3 x Height) - (50.3 x Age) + 26.9 #
• Female		(51.2 x Weight) + (24.5 x Height) - (207.5 x Age) + 1.629.8 #
**Muller [11]**	Healthy; obese; 5-17	
• Male		(50 x Weight) + (25.3 x Height) - (50.3 x Age) + 26.9 #
• Female		(51.2 x Weight) + (24.5 x Height) - (207.5 x Age) + 1.629.8 #
**Mifflin [12]**	Healthy; obese; 19-78	
		(9.99 x Weight) + (6.25 x Height) - (4.92 x Age) + (166 x sex (1 male; 0 female) - 161
**Lazzer (Equation 1) [13]**	Obese; 7-18	
		(12 x Weight) - (14 x Age) + (241 x Sex (1 male; 0 female)) + 909
**Caldwell-Kennedy [14]**	Healthy; obese; Studied in critically ill, mechanically ventilated [15]; 19-78	
		22 + (31.05 x Weight) + (1.16 x Age)
**White (Equation 2) [16]**	Critically ill, mechanically ventilated; 54±53 months	
		(17 x Age) - (48 x Weight) + (292 x Temperature (°C)) - 9677 #
**Meyer (Equation C) [17]**	Critically ill, mechanically ventilated; 3-16	
• *<3 years old*		(88 x Weight) + 92 - $(0.7 \times \text{Weight})^2$ - (37 x Weight)
• *3-10 years old*		(88 x Weight) + 110 - $(0.7 \times \text{Weight})^2$ - (37 x Weight)
• *10-18 years old*		(88 x Weight) - 910 - $(0.7 \times \text{Weight})^2$

*****Height in meters; # Predicted energy expenditure in KJ/day*

References

1. Harris, J.A.; Benedict, F.G. A Biometric Study of Human Basal Metabolism. *Proc Natl Acad Sci U S A* **1918**, *4*, 370–373, doi:10.1073/pnas.4.12.370.
2. Schofield, W.N. Predicting Basal Metabolic Rate, New Standards and Review of Previous Work. *Hum Nutr Clin Nutr* **1985**, *39 Suppl 1*, 5–41.
3. Energy and Protein Requirements. Report of a Joint FAO/WHO/UNU Expert Consultation. *World Health Organ Tech Rep Ser* **1985**, *724*, 1–206.
4. Henry, C.J.K. Basal Metabolic Rate Studies in Humans: Measurement and Development of New Equations. *Public Health Nutr* **2005**, *8*, 1133–1152, doi:10.1079/phn2005801.
5. Trumbo, P.; Schlicker, S.; Yates, A.A.; Poos, M.; Food and Nutrition Board of the Institute of Medicine, The National Academies Dietary Reference Intakes for Energy, Carbohydrate, Fiber, Fat, Fatty Acids, Cholesterol, Protein and Amino Acids. *J Am Diet Assoc* **2002**, *102*, 1621–1630, doi:10.1016/s0002-8223(02)90346-9.
6. Lawrence, J.C.; Lee, H.-M.; Kim, J.-H.; Kim, E.-K. Variability in Results from Predicted Resting Energy Needs as Compared to Measured Resting Energy Expenditure in Korean Children. *Nutr Res* **2009**, *29*, 777–783, doi:10.1016/j.nutres.2009.10.017.
7. Kaneko, K.; Ito, C.; Koizumi, K.; Watanabe, S.; Umeda, Y.; Ishikawa-Takata, K. Resting Energy Expenditure (REE) in Six- to Seventeen-Year-Old Japanese Children and Adolescents. *J Nutr Sci Vitaminol (Tokyo)* **2013**, *59*, 299–309, doi:10.3177/jnsv.59.299.
8. Dietz, W.H.; Bandini, L.G.; Schoeller, D.A. Estimates of Metabolic Rate in Obese and Nonobese Adolescents. *J Pediatr* **1991**, *118*, 146–149, doi:10.1016/s0022-3476(05)81870-0.
9. Maffeis, C.; Schutz, Y.; Micciolo, R.; Zoccante, L.; Pinelli, L. Resting Metabolic Rate in Six- to Ten-Year-Old Obese and Nonobese Children. *J Pediatr* **1993**, *122*, 556–562, doi:10.1016/s0022-3476(05)83535-8.
10. Molnár, D.; Jeges, S.; Erhardt, E.; Schutz, Y. Measured and Predicted Resting Metabolic Rate in Obese and Nonobese Adolescents. *J Pediatr* **1995**, *127*, 571–577, doi:10.1016/s0022-3476(95)70114-1.
11. Müller, M.J.; Bosy-Westphal, A.; Klaus, S.; Kreymann, G.; Lührmann, P.M.; Neuhäuser-Berthold, M.; Noack, R.; Pirke, K.M.; Platte, P.; Selberg, O.; et al. World Health Organization Equations Have Shortcomings for Predicting Resting Energy Expenditure in Persons from a Modern, Affluent Population: Generation of a New Reference Standard from a Retrospective Analysis of a German Database of Resting Energy Expenditure. *Am J Clin Nutr* **2004**, *80*, 1379–1390, doi:10.1093/ajcn/80.5.1379.
12. Mifflin, M.D.; St Jeor, S.T.; Hill, L.A.; Scott, B.J.; Daugherty, S.A.; Koh, Y.O. A New Predictive Equation for Resting Energy Expenditure in Healthy Individuals. *Am J Clin Nutr* **1990**, *51*, 241–247, doi:10.1093/ajcn/51.2.241.
13. Lazzer, S.; Patrizi, A.; De Col, A.; Saezza, A.; Sartorio, A. Prediction of Basal Metabolic Rate in Obese Children and Adolescents Considering Pubertal Stages and Anthropometric Characteristics or Body Composition. *Eur J Clin Nutr* **2014**, *68*, 695–699, doi:10.1038/ejcn.2014.26.
14. Caldwell, M.D.; Kennedy-Caldwell, C. Normal Nutritional Requirements. *Surg Clin North Am* **1981**, *61*, 489–507, doi:10.1016/s0039-6109(16)42432-1.
15. Vazquez Martinez, J.L.; Martinez-Romillo, P.D.; Diez Sebastian, J.; Ruza Tarrio, F. Predicted versus Measured Energy Expenditure by Continuous, Online Indirect Calorimetry in Ventilated, Critically Ill Children during the Early Postinjury Period. *Pediatr Crit Care Med* **2004**, *5*, 19–27, doi:10.1097/01.PCC.0000102224.98095.0A.
16. White, M.S.; Shepherd, R.W.; McEniery, J.A. Energy Expenditure in 100 Ventilated, Critically Ill Children: Improving the Accuracy of Predictive Equations. *Crit Care Med* **2000**, *28*, 2307–2312, doi:10.1097/00003246-200007000-00021.
17. Meyer, R.; Kulinskaya, E.; Briassoulis, G.; Taylor, R.M.; Cooper, M.; Pathan, N.; Habibi, P. The Challenge of Developing a New Predictive Formula to Estimate Energy Requirements in Ventilated Critically Ill Children. *Nutr Clin Pract* **2012**, *27*, 669–676, doi:10.1177/0884533612448479.
