# Supplementary figures and images for: External Validation of Equations to Estimate Resting Energy Expenditure in Critically Ill Children and Adolescents with and without Malnutrition: A Cross-Sectional Study
Source: Nutrients. 2022 Oct 6;14(19):4149. doi: 10.3390/nu14194149 (PMC9572704; doi:10.3390/nu14194149)

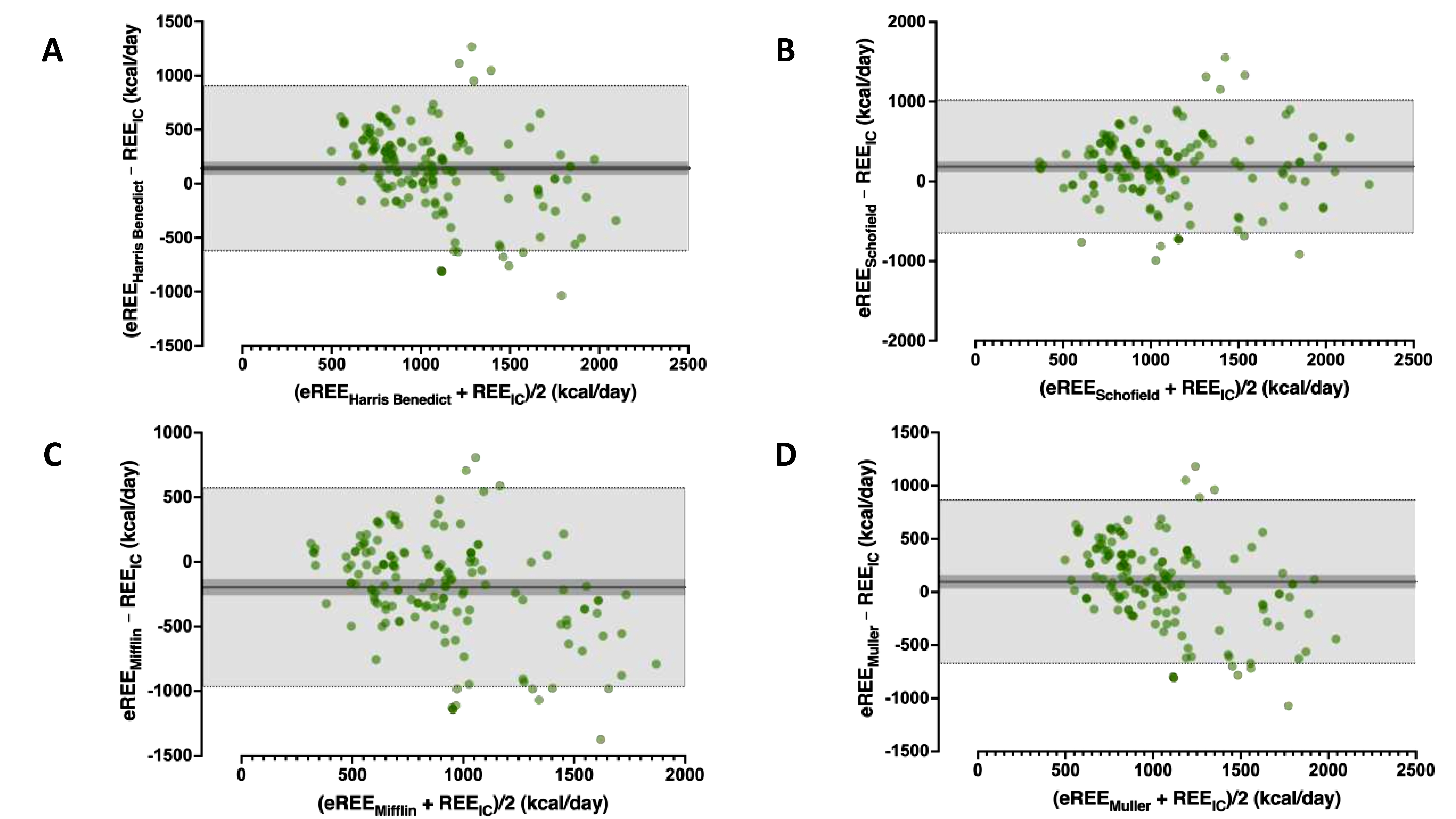

Supplement: Supplementary file 1 [file nutrients-14-04149-s001.zip › Figure 1S.tiff]

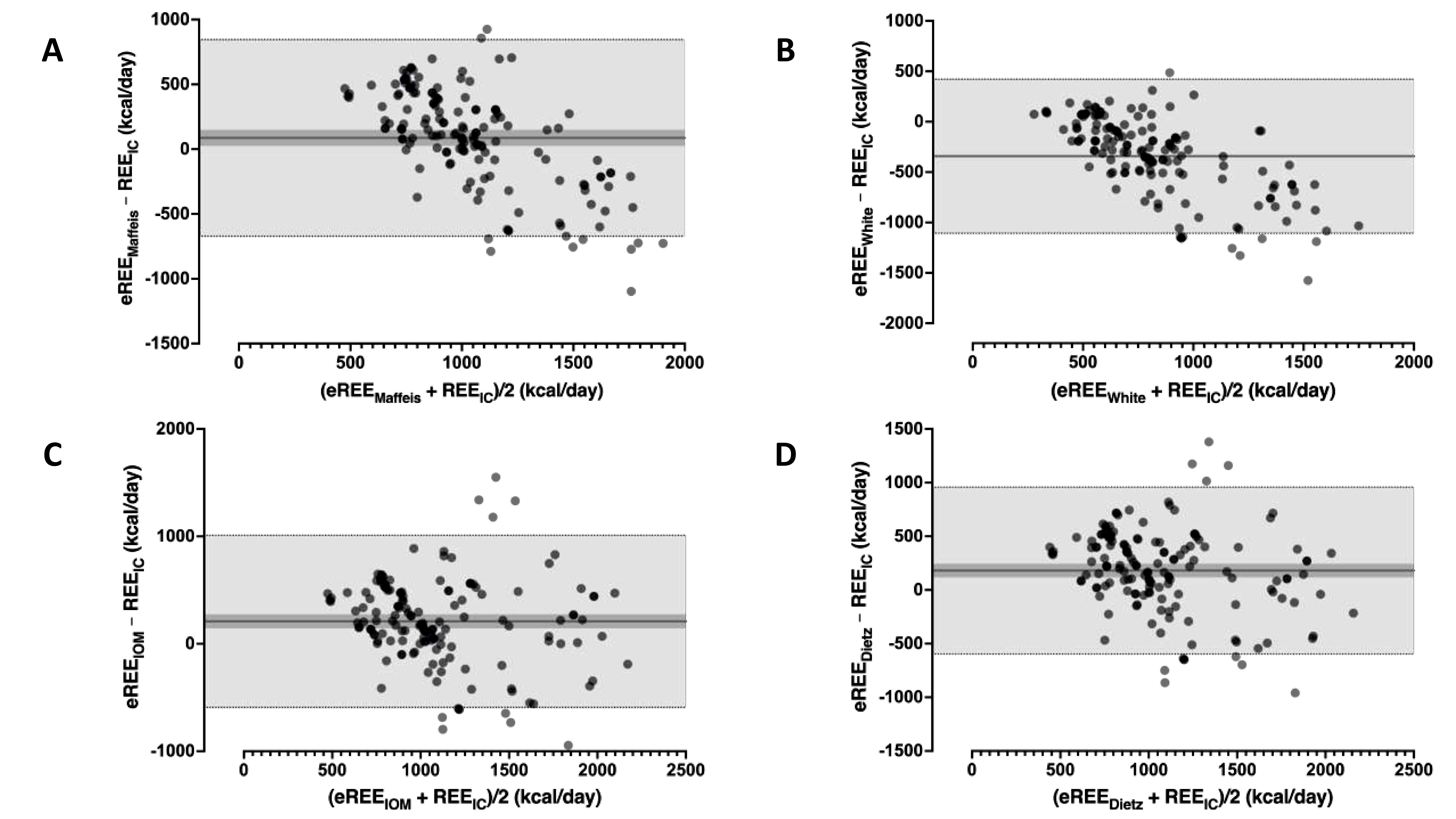

Supplement: Supplementary file 1 [file nutrients-14-04149-s001.zip › Figure 2S.tiff]

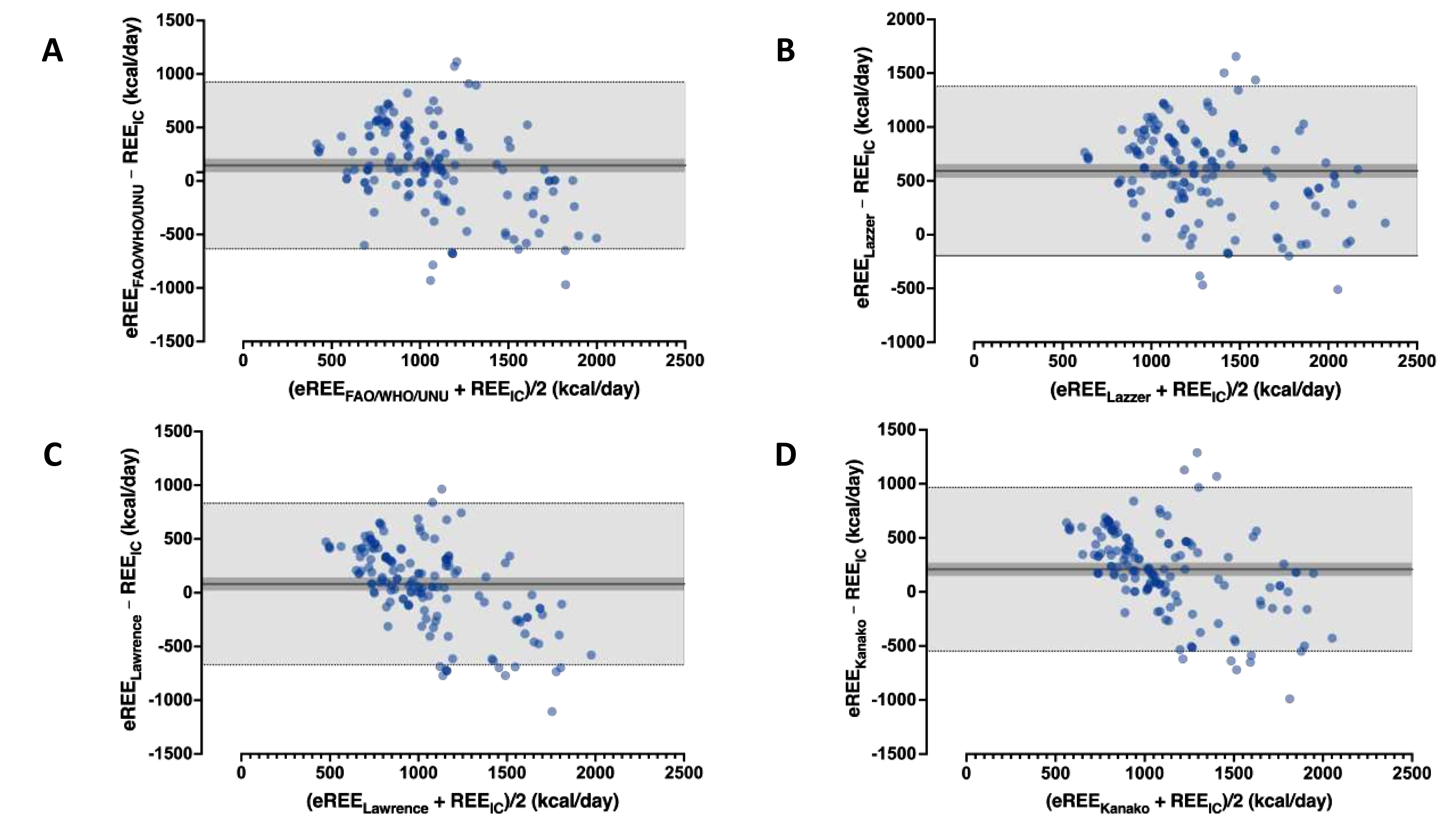

Supplement: Supplementary file 1 [file nutrients-14-04149-s001.zip › Figure 3S.tiff]
